# MicroRNAs in extracellular vesicles: Sorting mechanisms, diagnostic value, isolation, and detection technology

**DOI:** 10.3389/fbioe.2022.948959

**Published:** 2022-10-17

**Authors:** Dongjie Xu, Kaili Di, Boyue Fan, Jie Wu, Xinrui Gu, Yifan Sun, Adeel Khan, Peng Li, Zhiyang Li

**Affiliations:** ^1^ College of Animal Science, Yangtze University, Jingzhou, China; ^2^ Department of Laboratory Medicine, Affiliated Drum Tower Hospital, Medical School of Nanjing University, Nanjing, China; ^3^ Jiangsu Key Laboratory of Medical Science and Laboratory Medicine, School of Medicine, Jiangsu University, Zhenjiang, China; ^4^ State Key Laboratory of Bioelectronics, School of Biological Science and Medical Engineering, National Demonstration Center for Experimental Biomedical Engineering Education (Southeast University), Southeast University, Nanjing, China

**Keywords:** extracellular vesicles, microRNAs, liquid biopsy biomarkers, sorting mechanisms, diagnostic value, isolation and detection technology

## Abstract

MicroRNAs (miRNAs) are a class of short, single-stranded, noncoding RNAs, with a length of about 18–22 nucleotides. Extracellular vesicles (EVs) are derived from cells and play a vital role in the development of diseases and can be used as biomarkers for liquid biopsy, as they are the carriers of miRNA. Existing studies have found that most of the functions of miRNA are mainly realized through intercellular transmission of EVs, which can protect and sort miRNAs. Meanwhile, detection sensitivity and specificity of EV-derived miRNA are higher than those of conventional serum biomarkers. In recent years, EVs have been expected to become a new marker for liquid biopsy. This review summarizes recent progress in several aspects of EVs, including sorting mechanisms, diagnostic value, and technology for isolation of EVs and detection of EV-derived miRNAs. In addition, the study reviews challenges and future research avenues in the field of EVs, providing a basis for the application of EV-derived miRNAs as a disease marker to be used in clinical diagnosis and even for the development of point-of-care testing (POCT) platforms.

## 1 Introduction

**FIGURE 1:**
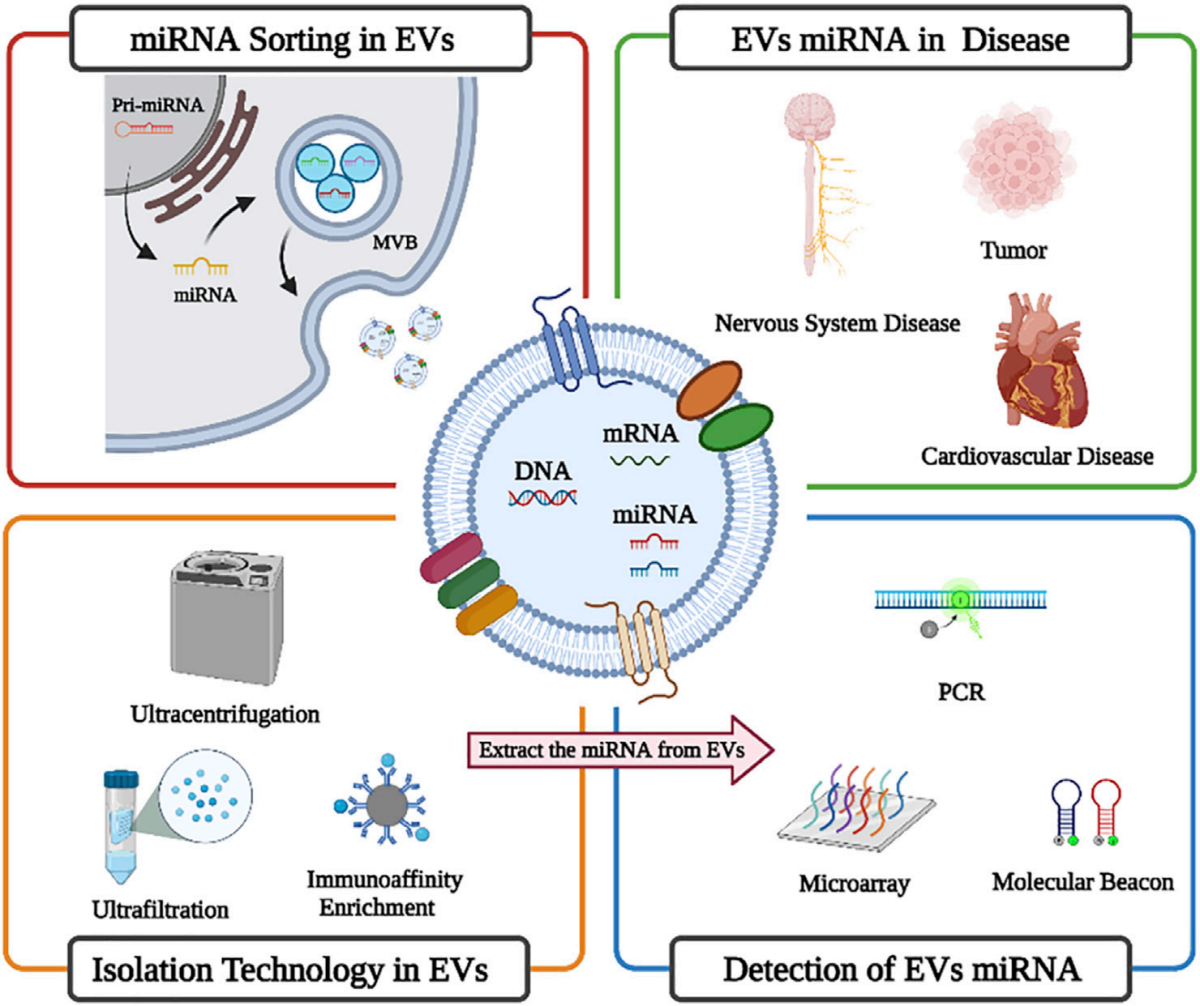
Brief overview of EV-derived miRNA sorting mechanisms, diagnostic value, isolation, and detection technology (created with BioRender.com).

Extracellular vesicles (EVs) are membrane-enclosed entities shed by majority of cells. EVs range in size, from 50 nm to 2 μm, and are abundant in almost all human body fluids. EVs are enriched with bioactive components such as protein, nucleic acid, and lipids. The enriched EVs and their content can specifically relate to the onset and prognosis of a plethora of diseases (Joyce et al., 2016; Bebelman et al., 2018; Momen-Heravi et al., 2018). EVs can also be used as a tumor detection marker for tumor (Xie et al., 2020a). MicroRNAs (miRNAs) are a class of short noncoding RNAs with a length of about 18–22 nucleotides that are widely produced by all eukaryotic cells (Friedman et al., 2009; Cortez et al., 2011). A large number of these miRNAs exist in body fluids, including plasma (Mitchell et al., 2008), urine (Bandini, 2021), saliva (Kamal et al., 2020), bronchoalveolar lavage fluid (Groot and Lee, 2020), amniotic fluid (Keller et al., 2011), and semen (Ruiz-Plazas et al., 2021). Circulating miRNAs are stable in body fluids and can be protected by binding to argonaute (AGO) proteins, high-density lipoprotein (HDL), and/or encapsulated in EVs (Wagner et al., 2013; Qu et al., 2016; Groot and Lee, 2020). The microRNA can control protein expression by binding to mRNA (Song et al., 2018; Gui et al., 2021; Ferri et al., 2022). The EVs from diseased sources have been proven to have unique miRNA expression profiles. In addition, specific miRNA expression characteristics not only reflect the presence of diseases at early stages but can also reflect the dynamic development of diseases at late stages, as well as diseases prognosis and drug resistance (Hu et al., 2010). With maturity of miRNA from EV research, miRNA is gradually employed for clinical diagnosis. For instance, about seven kinds of miRNA detection kits are available for diagnosing early-stage hepatocellular carcinoma (HCC) based on a plasma miRNA panel found by the Jia Fan group. Similarly, pancreatic cancer miRNA-25 detection kits have also obtained China Food and Drug Administration (CFDA) approval (Zhou et al., 2011). Herein, we mainly introduce EV-derived miRNA sorting mechanisms, diagnostic value, isolation, and detection technology (Figure 1).

## 2 Mechanisms for microRNA sorting in extracellular vesicles

**FIGURE 2:**
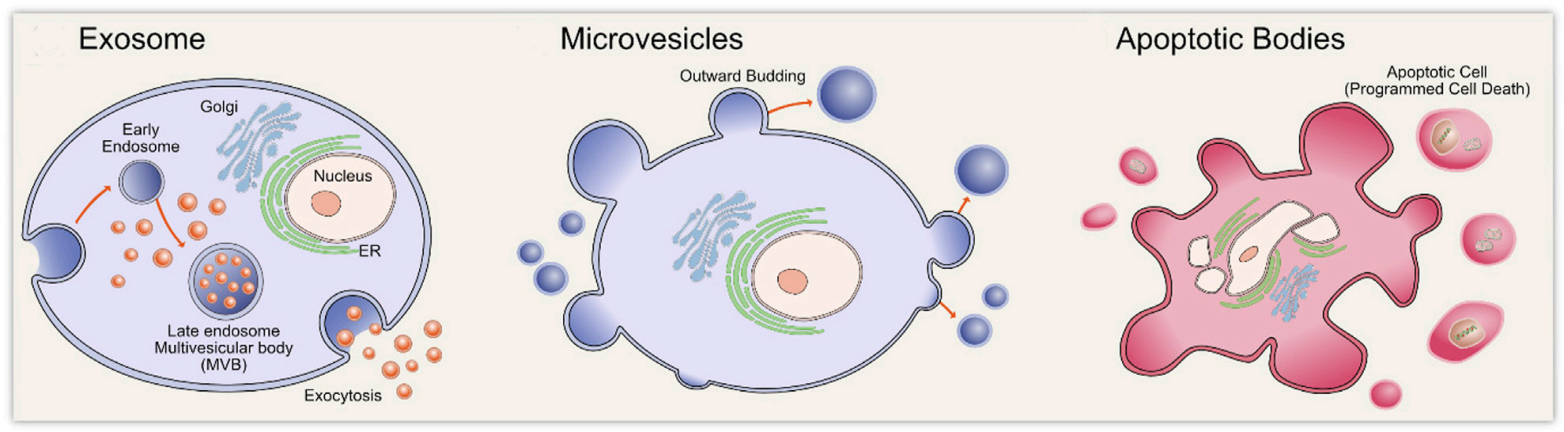
Diagrammatic overview of the three types of extracellular vesicles biogenesis including exosomes biogenesis, microvesicles biogenesis, and apoptotic bodies biogenesis. Reprinted with permission from Gurunathan et al. (2019).

**FIGURE 3:**
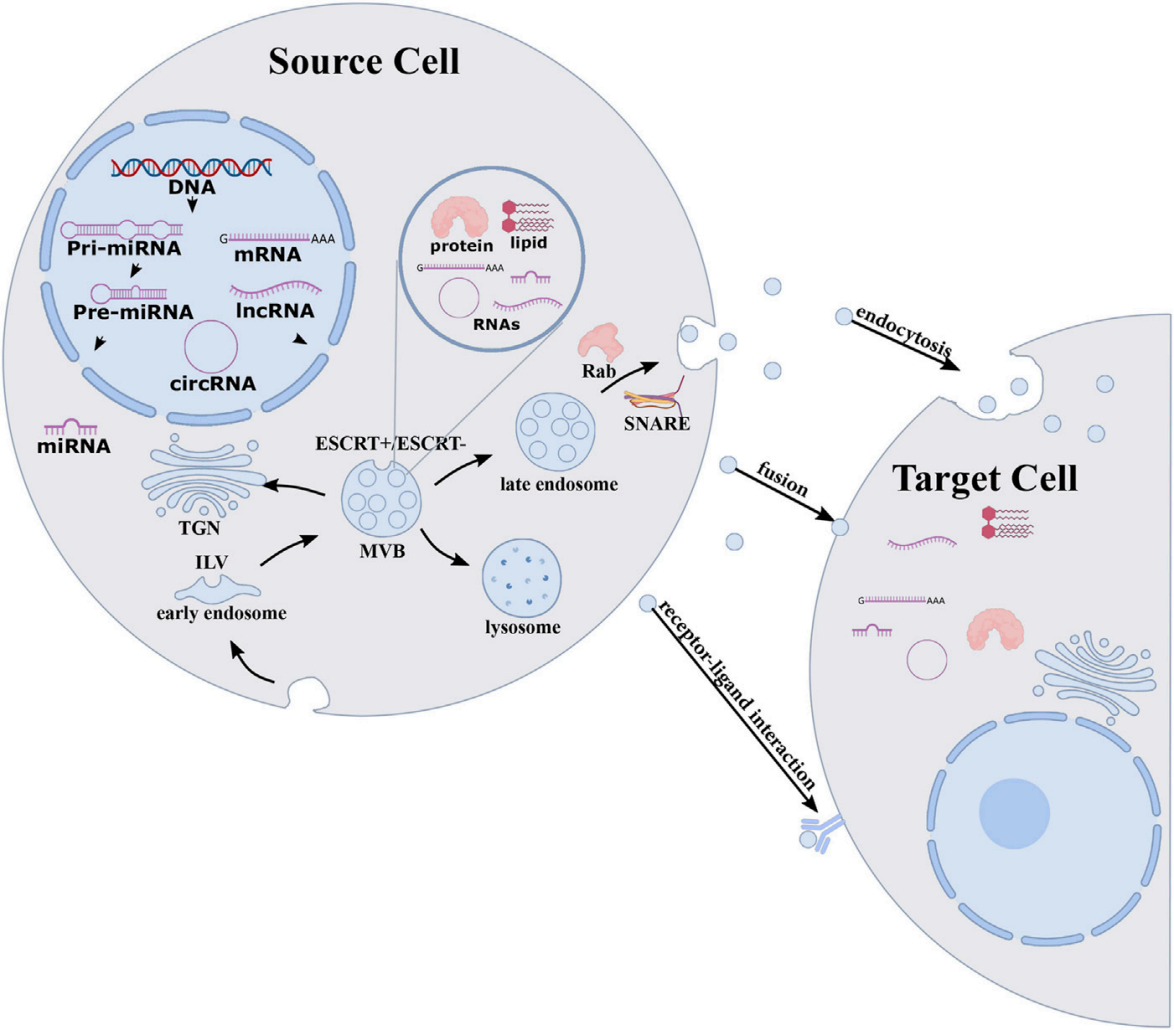
sorting mechanism for exosomal miRNAs. MiRNA originates in nucleus and enzymatically processed to form mature miRNA that are loaded into the MVBs through endosomal sorting complex required to transport ESCRT-dependent or ESCRT-independent pathways. MVBs of endosomes migrate to the plasma membrane and fuse, causing exosome and exosomal cargoes to be released into the extracellular space and delivered to target cells. Reprinted with permission from Yue et al. (2020).

Most prokaryotic and eukaryotic cells rain EVs. EVs can be divided into three types on the basis of their biogenesis (Figure 2). Exosomes, with a diameter of about 30–150 nm, originate in the multivesicular body (MVB) of endosomes, which subsequently migrate to the plasma membrane and fuse, causing exosomes to be released into the extracellular space. Microvesicles of about 100–1,000 nm diameter are formed from direct outward germination of the plasma membrane. Apoptotic bodies with a 50 nm–2 μm diameter are formed from cell apoptosis (Cocucci and Meldolesi, 2015; Thery et al., 2018; Gurunathan et al., 2019; Yuan and Li, 2019; Kalluri and LeBleu, 2020). They contain different types of biomolecules, including proteins, lipids, and nucleic acids. These substances can be protected by the EV membrane structure and thus transported to distant cells (Yanez-Mo et al., 2015; Jan et al., 2019; Mathieu et al., 2019). EVs have been shown to have multiple biological functions, including immune response, antigen presentation, and intracellular communication (Lasser et al., 2011; Tomasetti et al., 2017; Mathieu et al., 2019). EVs are enriched with noncoding RNAs, including miRNAs, tRNA, rRNA, mitochondrial RNA, circular RNA (circRNA), and long noncoding RNA (lncRNA) (Michaela and Aigner, 2021; Yu et al., 2022). Extracellular miRNA mainly exists in the EVs (Zhang et al., 2015), and although the detailed mechanisms by which the miRNAs are packaged into the EVs are largely unknown, they are thought to be selectively classified into the EVs (Turchinovich et al., 2013; O'Brien et al., 2020; Makarova et al., 2021). Both miRNA and pre-miRNA can be secreted into exosomes and microvesicles in protein-bound and protein-free forms. Exosomal miRNAs are secreted by various pathways, including sequence-dependent or sequence-independent classification of miRNAs into MVB. Usually, the miRNA begins in the nucleus and is processed by enzymes (Figure 3) and transported to the outside of the cell through exosomes (Lee et al., 2012; Sun et al., 2018; Yue et al., 2020). Various studies have shown that there is more than one mechanism for miRNA sorting (Montecalvo et al., 2012; Kosaka et al., 2013; Tran, 2016). For example, Kosaka et al. (2013) demonstrated that neurilemphospholipase 2 (nSMase2) can regulate the secretion of exosome miRNA and promote angiogenesis and metastasis in the tumor microenvironment. Montecalvo et al. (2012) demonstrated that dendritic cells (DC cells) release exosomes with different miRNAs according to degree of maturity, and they found that exosomes release miRNAs into the cytoplasm after fusion with target DC cells. MiRNAs derived from EVs perform different functions in different target cells, such as miR-21, which is associated with senescent fibrosis of the kidneys (Liu et al., 2020) and is also highly expressed in breast cancer cells (Lee et al., 2019). Therefore, more research is still needed to reveal the generation mechanism and biological impact of EV-derived miRNAs.

## 3 Diagnostic value of extracellular vesicle-derived microRNA in diseases

MiRNA is correlated with the occurrence and development of many diseases (Peng et al., 2020), including tumor, neurological diseases, cardiovascular diseases, and many other diseases. The EV-derived miRNAs have many unique advantages as biomarkers (Schwarzenbach, 2017; Sanz-Rubio et al., 2018). First, the expression profile of EV-derived miRNA is similar to that of cells from which it originates (Chaput and Thery, 2011). MiRNAs are transported to recipient cells through EVs to achieve regulatory functions (Skog et al., 2008). Second, many EVs exist in various biological body fluids, which is convenient for sampling and screening diseases (Cai et al., 2015). Third, the EV lipid membranes protect miRNA from degradation and remain stable in body fluids (Yu et al., 2021). Moreover, EV-derived miRNA is considered highly sensitive and specific diagnostic tools for numerous diseases (Jin et al., 2017).

### 3.1 Extracellular vesicle-derived microRNA in tumor

The ability of tumor to proliferate and metastasize is related to the intercellular communication mediating in tumor microenvironment (TME) (Chiarugi and Cirri, 2016; Tian et al., 2019; Tan et al., 2020). The EVs secreted by cancer cells can mediate intercellular communication and are mediators of key signals in TME, playing an essential role in realizing intercellular material transport and information transmission in TME (Bao et al., 2018; Harmati et al., 2019; Xie et al., 2020b; Yekula et al., 2020). The EVs derived from tumor cells can participate in the proliferation and migration of tumor cells by activating a variety of signaling pathways, changing the physiological state of target cells and affecting the tumor microenvironment (Qu et al., 2020). MiRNA encapsulated in EVs can also enhance tumor invasiveness and metastasis (Mao et al., 2018a; Bao et al., 2018; Ludwig et al., 2020).

In a variety of tumor diseases, such as bladder cancer (Piao et al., 2021), ovarian cancer (Taylor and Gercel-Taylor, 2008), non-small-cell lung cancer (Wu and Shen, 2020), breast cancer (Volovat et al., 2020), prostate cancer (Sobhani et al., 2020), pancreatic cancer (Savareh et al., 2020), and gastric cancer (Cheng et al., 2020), the levels of miRNA in the EVs obtained from plasma of tumor patients were significantly higher than those of healthy controls. Certain disease-related miRNAs are selectively incorporated into the EVs, with significant differences in the miRNAs content compared to cells (Baglio et al., 2015). miRNA-224 enclosed in EVs is the diagnostic and prognostic marker for hepatocellular carcinoma (Cui et al., 2019). Zhou et al. (2017) identified six miRNAs (miR-19b-3p, miR-21-5p, miR-221-3p, miR-584-5p, miR-425-5p, and miR-409-3p) differentially expressed in exosomes from patients’ plasma with lung adenocarcinoma, which can distinguish patients with lung adenocarcinoma from healthy individuals. Jin et al. (2017) found that the expression of let-7b-5p and miR-486-5p in EVs could distinguish between non-small-cell lung cancer (NSCLC) patients and healthy individuals rather than in the supernatant without EVs. These studies have shown that the use of EV-derived miRNAs in serum as biomarkers has exceptional high stability and specificity (Moloudizargari et al., 2021) and can be used for the early detection, prognosis, and monitoring of cancer.

### 3.2 Extracellular vesicle-derived microRNAs in cardiovascular disease

EV-derived miRNA plays an important role in the occurrence and progression of cardiovascular diseases (Hergenreider et al., 2012; Murugesan et al., 2021; Zhang and Huang, 2021), such as atherosclerosis, acute coronary syndrome, heart failure (HF), myocardial ischemia-reperfusion injury, and pulmonary hypertension (Matkovich et al., 2009; Tian et al., 2017; Kalayinia et al., 2021; Zheng et al., 2021). Ren et al. (2020) found that the EVs from adventitial fibroblasts (AFs) regulate vascular smooth muscle cell (VSMC) proliferation by transporting miR55-5P and angiotensin converting enzyme (ACE), which plays a vital role in vascular remodeling.

EV-derived miRNA-30 members are key modulators of complex biological processes in a variety of cardiovascular diseases, including ischemic heart disease, heart failure, hypertension, and arrhythmias. They are attractive diagnostic and prognostic biomarkers in the cardiovascular field (Mao et al., 2018b). Studies have shown that the EV-derived miRNA (miR-17-5p, miR-126-5p, and miR-145-3p) can be of diagnostic value for acute myocardial infarction (AMI), and a combination of three miRNAs can improve the area under curve (AUC) and receiver operating characteristic curve (ROC) values, providing higher accuracy in the diagnosis of AMI (Xue et al., 2019; Mir et al., 2021).

### 3.3 Extracellular vesicle-derived microRNAs in nervous system diseases

At present, the pathogenesis of inflammatory diseases of the central nervous system [including neurodegenerative diseases (Ruan et al., 2021; Xiao et al., 2021)] and autoimmune diseases (Xu et al., 2022) has not been completely clarified, but more and more studies have begun to focus on the role of EVs in autoimmune diseases and research on treatment of EVs (Guo et al., 2019; Chu et al., 2020; Dar et al., 2021; Saeedi et al., 2021; Xia et al., 2021). Studies have found that multiple miRNAs are involved in neuroinflammation and neurological diseases, such as Parkinson’s disease (Xie et al., 2020c), Alzheimer’s disease (AD) (Dong et al., 2021), amyotrophic lateral sclerosis (Liu et al., 2022), and depression (Trotta et al., 2018; Chen and Wang, 2021). The blood–brain barrier (BBB) is the barrier between plasma and brain tissue, which can prevent harmful substances from entering the brain tissue from blood (He et al., 2018). The EVs act as transporters for miRNAs that cross the endothelial layer of the BBB, facilitating communication through biological fluids between the brain and distant organs (Montecalvo et al., 2013; Xia et al., 2021). Neurons and neurological cells release EVs, and their cargo can function in cellular communication and neuroinflammation by delivering mRNAs, miRNAs, and proteins (Gupta and Pulliam, 2014; Pegtel et al., 2014; Markoutsa et al., 2022). The EVs have been identified as a potential cell-to-cell carrier of misfolded proteins associated with neurodegenerative diseases in the central nervous system in most neurodegenerative diseases, and Aβ peptide, viral protein, alpha synuclein, tau, and superoxide dismutase (SOD) pathogenic aggregates are released from cells as exosomes (Saman et al., 2012; Schneider and Simons, 2013). These proteins form aggregates (amyloid and ion-like proteins) that escape normal degradation mechanisms. Cerebrospinal fluid (CSF) reflects physiological and pathological changes in the brain and is the best indicator for most central nervous system diseases, but sampling difficulties are unsuitable for disease screening. Compared with CSF, blood is easier to obtain, thus avoiding the invasive process of sample collection, and it is more suitable as a screening marker for neurological diseases. Moreover, Cheng et al. (2015) performed an unbiased next-generation sequencing (NGS) analysis of serum EV-derived miRNAs, identifying an AD-specific 16-miRNA signature that differ between healthy and AD patients, and used a random forest model to predict clinical classification with 87% sensitivity and 77% specificity (Lv et al., 2013).

### 3.4 Extracellular vesicle-derived microRNAs in kidney diseases

Recent studies have shown that the EV-miRNAs play an important role in kidney diseases and can also be a valuable biomarker for diagnosis. It was found that miR-29c in EVs can be related to renal function, and there are differences in the expression of EVs-miR-29c in patients with varying degrees of renal fibrosis (Lv et al., 2013). Similarly, miR-200b in nonproximal tubulo-derived urinary exosomes is expressed differently in normal people and patients with renal fibrosis, which can be used as a biomarker for renal fibrosis and can replace traditional invasive renal biopsy for diagnosis of renal fibrosis (Yu et al., 2018). It has been validated by sequencing and qPCR of EV-derived miRNAs that significant differences exist in the expression of miR-215-5p, miR-378i, miR-365b-3p, and miR-135b-5p in the urinary exosomes of immunoglobulin A nephropathy patients and healthy controls (Min et al., 2017). MiR-18a-5p was highly expressed in the acute phase of injury, and miR-132-3p was upregulated during the transition between acute and fibrotic injury; miR-146b-5p is highly expressed in the high fibrosis phase (Pellegrini et al., 2015). These studies suggest that miRNA has the potential to act as markers for kidney disease.

### 3.5 Extracellular vesicles-derived microRNAs in liver diseases

EVs can be produced by all types of cells in the liver, are involved in transmitting information between liver cells, and the EVs produced by liver cells are loaded with miRNAs that can be used in diagnosing liver-related diseases. Another research group verified by a mouse model of alcoholic hepatitis that miR-192, miR-122, miR-30a, miR-744, miR-1246, miR-30b, and miR-130a were elevated in alcohol-fed mice. Further testing was then carried out in human patients yielding results consistent with animal models showing that the EV-miRNAs are potential biomarkers of alcoholic hepatitis (Momen-Heravi et al., 2015). Elevated levels of miR-122 and miR-192 in EVs were found in models of nonalcoholic fatty liver mice, which could be used as a promising diagnostic marker (Newman et al., 2022). Another group found that higher expression of miR-19a in exosomes was also observed in serum from hepatocytes and patients with chronic hepatitis C virus (HCV) fibrosis infected with HCV compared with healthy volunteers and patients with non-HCV-associated liver disease (Devhare et al., 2017). These studies help us understand the role of EV-miRNAs in the development of liver disease.

## 4 Advances in isolation technology of microRNAs in extracellular vesicles

There are many miRNA binding to free proteins in that body fluid environment; therefore, for detection of EV-derived miRNAs, it is necessary to isolate the EVs first and then use TRIzol and other methods to isolate and purify EV-derived miRNAs (Hu et al., 2018; Yang et al., 2018). These steps take much time, and it is imperative to realize the timely detection of miRNA. Herein, we first introduce the purification technology for EV and then discuss the miRNA purification technology.

### 4.1 Progress of extracellular vesicle isolation and purification technology

The isolation method for EVs is very important for subsequent analysis of EV miRNA. Currently, there are many isolation methods for EVs. However, ultracentrifugation (UC), density gradient centrifugation (DGC), size exclusion chromatography (SEC), co-precipitation, and immunoaffinity enrichment are the most widely used methods. The summary of the contemporary methods for EV isolation is as follows.

#### 4.1.1 Ultracentrifugation

A differential ultracentrifugation method was proposed by Dr. Thery in France for the separation of EVs based on physical and chemical properties of EVs (Thery et al., 2006). It is considered a classical (Momen-Heravi, 2017) and gold standard EV isolation method (Royo et al., 2020). Most researchers isolate EVs by UC (Gardiner et al., 2016). The differential ultracentrifugation method is mainly used to separate the EVs from other components by continuously increasing centrifugal force on samples and removing cells and cell fragments. Many studies have proved its reliability. The usually used ultracentrifugation speed is 110,000 g for centrifugal force to centrifuge the sample twice for 70 min and finally blow and mix into the buffer (Lobb et al., 2015). Although EV researchers have widely used differential ultracentrifugation, it still has many drawbacks. First, this method requires expensive instruments. Second, centrifugation can easily lead to EV aggregation and will be mixed with non-EV impurities, such as protein polymers and viruses. Especially in sticky body fluids such as plasma, there are more impurities. Third, the high-speed process of ultracentrifugation will cause damage to EVs to a certain extent, and the longtime of ultracentrifugation may lead to EV morphological changes (Issman et al., 2013). The EVs extracted by UC have high purity and are conducive to downstream detection, but due to limitations of current instrumentation and extraction efficiency, it is difficult to use the separation of large number of clinical specimens’ samples, which is mainly used for researchers’ research. Density gradient centrifugation (DGC) and UC have similar principles in isolating the EVs; DGC can maintain the integrity of EVs, but it still has the corresponding disadvantages of UC, whereas the DGC is to form a specific density gradient medium in a centrifuge tube. At present, sucrose and iodoxanol are commonly used, and the density of medium increases from top to bottom. Under a certain centrifugal force, the smaller the density, the higher the distribution. Finally, the particles of different densities will stagnate in the corresponding iso density area. Density gradient centrifugation has a higher resolution and is often used to separate EVs with higher purity (Ashley et al., 2018). Some HDLs can still be co-separated, although density gradient centrifugation has a higher purity for EVs than ultrafast centrifugation (Yuana et al., 2014; Greening et al., 2015). DGC can isolate higher purity EVs, but it introduces exogenous components and has the same disadvantages as UC, which is not conducive for extraction of large clinical samples and detection of miRNAs.

#### 4.1.2 Size exclusion chromatography

**FIGURE 4:**
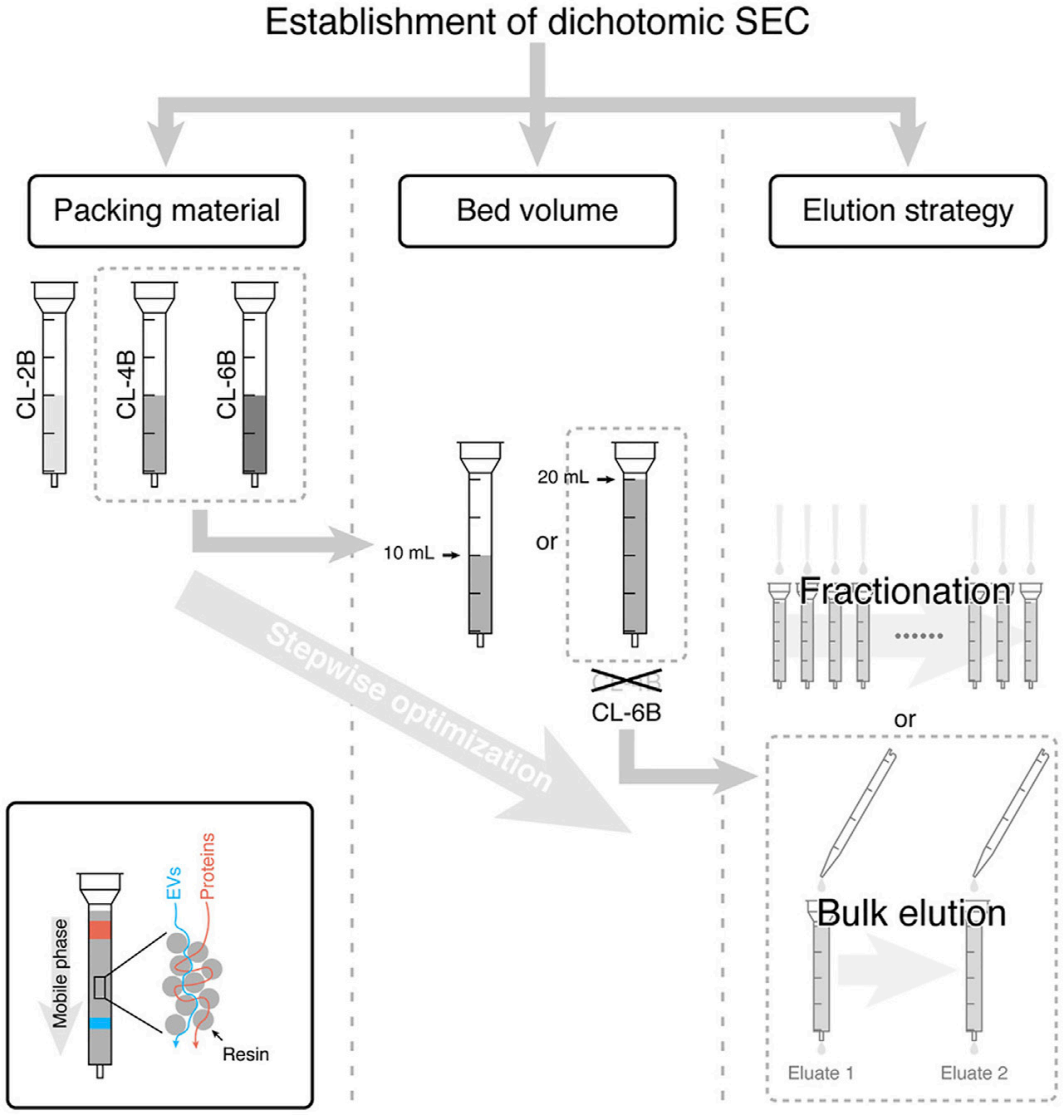
SEC-based EV isolation workflow and the underlying principle. In this study, CL-6B was demonstrated to be the best one from three Sepharose CL resins with the bed volume of 20 ml, and two bulk elution steps were sufficient for isolation of EVs. Reprinted with permission from Guo et al. (2021).

The SEC separation method is based on the particle size. Each molecule can pass through the hole in the polymer bead according to its size on a chromatographic column with a special pore size matrix. Molecules with smaller radius can enter the hole, and the elution speed is slow. Molecules with larger radii cannot enter the hole and move through the column more quickly. Most of the EVs can be eluted before soluble components (Boing et al., 2014). The size of interception aperture depends on the choice of the exclusion matrix. For example, with an aperture of about 60 nm for agarose 2B, SEC can remove 99% of soluble plasma proteins and > 95% of HDL without causing EV aggregation, preserving its integrity and biological activity (Boing et al., 2014). Plasma proteins are difficult to remove by differential ultracentrifugation or precipitators, which is simpler and less time-consuming than density gradient centrifugation. However, the SEC removes most of the overabundant soluble plasma proteins (Monguio-Tortajada et al., 2019). Moreover, some non-EV components mainly particles above the interceptor aperture, including viruses, protein polymers, and some large proteins such as chylomicrons, are also separated together. Koh et al. (2018) used ultracentrifugation and SEC to enrich the EVs from the plasma for clinical application. Guo et al. (2021) established a simplified dichotomic SEC method that only requires two bulk elutions to get the EVs in the Eluate 1 and proteins in the Eluate 2. This dichotomic SEC has fascinating potential for EV preparation for clinical testing and/or basic research (Figure 4).

#### 4.1.3 Ultrafiltration

The ultrafiltration method is simple and efficient and does not affect the biological activity of EVs (Taylor and Shah, 2015). This size-based separation method involves the use of a membrane filter with a specific pore size, such as a membrane filter with a typical pore size of 0.22 µm to collect EVs from the filtrate. For the sample to pass through the filter, negative pressure or centrifugal force is usually used as pressure. Ultrafiltration has a short time and a high recovery rate, effectively concentrating the EVs in large samples such as the culture medium (Haraszti et al., 2018). We have made a small extraction and filtration device for rapid extraction of EVs (Li et al., 2019). First, negative pressure was applied to remove large membrane fragments through a filter membrane with a pore size of 600 nm, and then the filtrate was used to remove small impurities through a filter membrane with a pore size of 20 nm. Chen et al. (2021) proposed a new ultrafiltration strategy: the exosome detection method *via* an ultrafast isolation system (EXODUS), which introduced dual coupled harmonic oscillations into a dual-membrane filter configuration to generate s-waves. The inhibition of the scaling effect improved the treatment speed, yield, and purity and helped achieving nonblocking and ultrafast purification of EVs. Ultrafiltration and a high recovery rate can effectively concentrate EVs in large volume samples. However, this method requires complex equipment and is not easy to be popularized. Ultrafiltration only separates the EVs by size and lacks specificity; therefore, it cannot remove many impurities with similar particle sizes to EVs. When passing through the filter membrane, the EVs are stocked on the filter membrane, resulting in an inevitable loss.

#### 4.1.4 Co-precipitation

Recently, the polymer co-precipitation method, which changes the solubility of exosomes and precipitates them (Rider et al., 2016), has attracted many researchers’ attention for its simple and rapid use. Commercial kits based on polymer co-precipitation (e.g., ExoQuick and total exosome isolation) have been developed for EV extraction and isolation. Once the reagents are introduced, the solubility of EVs is significantly reduced and easily precipitated. Under the action of low centrifugal force, the precipitated EVs can be easily separated, thus avoiding the time-consuming centrifugal operation. The precipitation method can save more time, and the concentration of exosomes is 2.5-fold higher than ultracentrifugation (Coughlan et al., 2020). The total exosome isolation kit is commonly used for serum EV separation as an example. After mixing 30 μl of precipitant with 100 μl of serum sample, the sample is incubated at 4°C for 30 min and centrifuged at 10,000 g for 5 min to precipitate EVs. However, this method is not conducive to large-scale clinical application and may co-precipitate many organelle-related proteins, such as endoplasmic reticulum and lysosome, which is not conducive to a downstream analysis (Li et al., 2021). In addition, as additional components need to be added to the sample during extraction, the collected EVs cannot be directly used for subsequent cell culture or transport, limiting the application of EVs. The EVs is isolated by co-precipitation and will be mixed with protein impurities and require centrifuges. The method is not conducive to extraction of a large number of clinical specimens and miRNA detection.

#### 4.1.5 Immunoaffinity enrichment

The specificity and simplicity of immunoaffinity capture is an attractive method. To enrich EVs, antibodies specific to EV specific markers, such as CD9, CD63, CD81, epidermal growth factor receptor (EGFR), and epithelial cell adhesion molecule (EpCAM), are fixed on the surface of various vectors, such as an ELISA plate, and a magnetic bead or chip (Choi et al., 2021). Taking the exosome CD63 isolation kit (Life Technologies) as an example, the magnetic beads coated with anti-CD63 monoclonal antibodies are mixed with the sample and then incubated at 2–8°C for 18–22 h. After magnetic separation, the magnetic beads are cleaned with a washing buffer to remove the impurities, and the magnetic beads enriched with EVs could be directly used for flow cytometry detection (Oksvold et al., 2015). The immunoaffinity capture method can isolate specific subgroups of EVs, obtain high purity EVs, and have many advantages, such as simple operation. However, the incubation time between magnetic beads and samples is more extended, usually taking hours or even overnight. In addition, due to the strong affinity of antigen–antibody binding, it is challenging to separate immune magnetic beads from the EVs, which is not conducive to a subsequent functional analysis. Moreover, this method can only extract the EVs subgroup from the corresponding antigen, which is accompanied by the risk of bias. In combination with other labeling methods, such as fluorescent labeling, it can be used for the detection of EVs. It is an isolation method that is expected to be used for clinical applications. However, this method can only specifically enrich the EVs corresponding high antigen expression, which may cause the loss of EV-related miRNA information.

#### 4.1.6 Field flow fractionation

Field flow fractionation (FFF) is a kind of technology that can separate and characterize different size particles. Unlike size exclusion chromatography, FFF separation is performed in a single phase. The samples flow in the FFF channel in a parabolic form. Particles have the highest velocity in the center, and velocity decreases as they get closer to the channel wall. Zhang and Lyden (2019) successfully isolated EVs of different sizes from cell supernatant using the asymmetric flow field flow separation (AF4) technique. The particles in the sample first gather to one side of the channel under the action of an external field. The particles will remain at different distances from the tube wall due to their size, and smaller particles farther from the side wall flow faster and are thus eluded earlier than larger particles. This way, the EVs of different sizes can be obtained at specific periods. The sample preparation process of the asymmetric flow–flow separation method is cumbersome, resulting in low flux. The high initial sample concentration requirement and time-consuming operation also limit its wide application. This method has less damage to EVs and can sort out the EVs of different particle sizes. Some proteins of the same particle size may be adulterated. In studying EVs of different size, it has a potential application value.

#### 4.1.7 Acoustic-based isolation method

**FIGURE 5:**
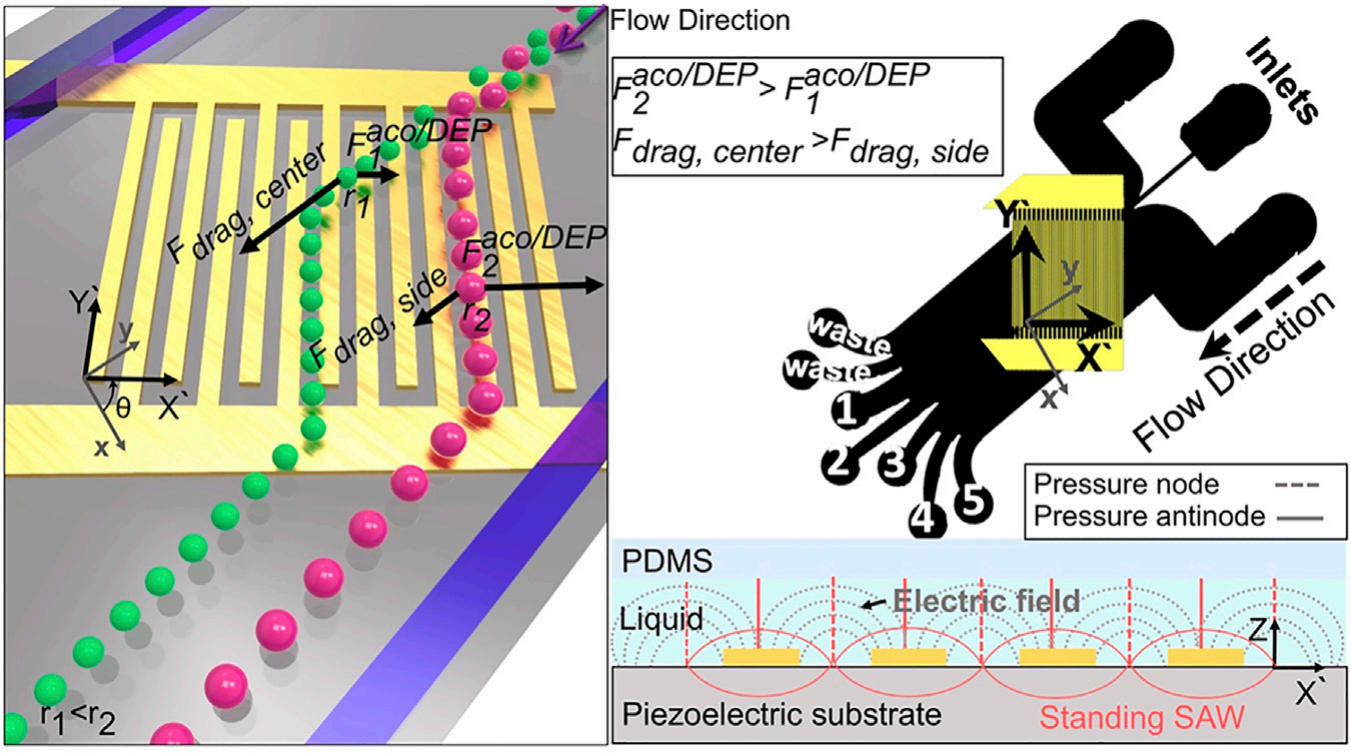
Schematic illustrates the working mechanism of the system. Left image shows that particles of different sizes are subjected to different forces in the acoustic and electric fields, causing the separation of particles of different sizes. The right one is the schematic diagram of PDMS microchannel device and the side view of electric and acoustic field around the electrodes (Tayebi et al., 2021).

Acoustic waves have high precision and biocompatibility in processing cells and other biological particles and can be used in combination with microfluidic technology for EV separation (Lee et al., 2015b). The acoustic-based microfluidics EV separation method typically uses ultrasonic waves to apply to particles in a sample (Huang et al., 2021). Under sound pressure, the particles are separated according to their physical properties, such as size and density, under different forces (Iliescu et al., 2019; Hassanpour Tamrin et al., 2021). Ku et al. (2018) captured the EVs by scattering between the microparticles in the resonance cavity and EVs by an ultrasonic wave. The integrated acoustic device is capable of fast operation, label-less, contactless, and continuous separation of EVs. Researchers also developed an integrated acoustic device that can directly separate the EVs from blood samples (Wu et al., 2017). The device combines acoustic wave and microfluidic technology into two separate modules. The first module can remove substances larger than 1 µm in diameter, such as cells, and the second module can separate larger microvesicles and apoptotic bodies from exosomes. By adjusting the input power and flow rate, the interception size of the two separation modules can be adjusted to flexibly adjust the obtained particle size, and the application range is more expansive. Tayebi et al. (2021) demonstrated a method for sorting the exosomes (<200 nm) and microvesicles (>300 nm) using both electrical and acoustic forces, with EV purification resulting in more than 95% purity and 81% recovery (Figure 5).

#### 4.1.8 Metallic oxide-based isolation method

**FIGURE 6:**
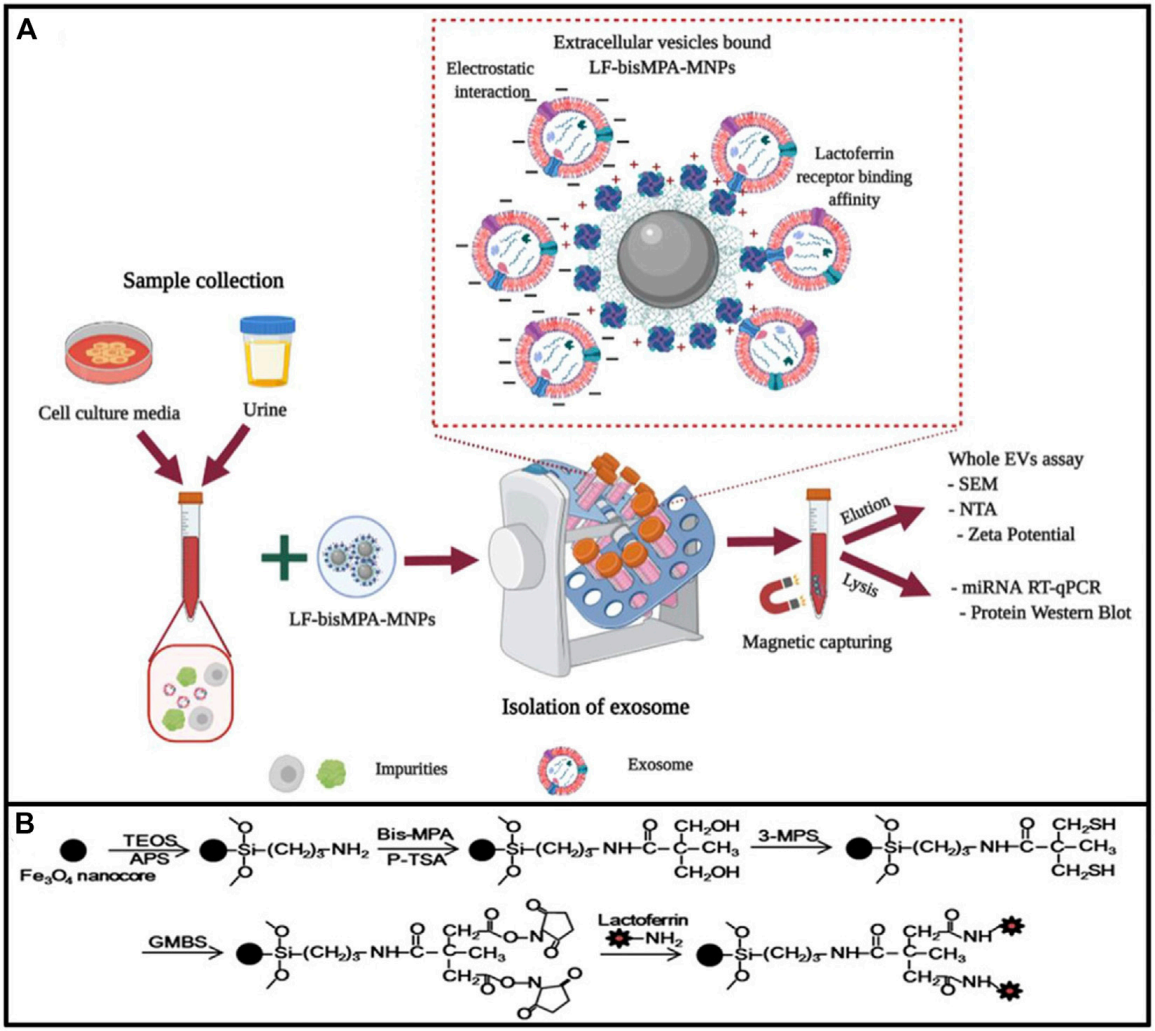
Diagrammatic elucidation of EV isolation by LF-bis-MPA-MNPs. **(A)** LF-bis-MPA-MNPs mediated EV isolation from cell culture media (CCM) and human urine. **(B)** Elucidation of LF-bis-MPA-MNPs synthesis and modification principal. Reprinted with permission from Dao et al. (2022).

Titanium dioxide (TiO_2_) can combine with phosphorylated amino acid residues including serine, tyrosine, and threonine (Liu et al., 2015). Under acidic conditions, the surface of the TiO_2_ beads is cationic and specifically binds to the phosphate groups on phosphorylated modified peptide fragments. TiO_2_ materials have been extensively studied in the research field of phosphorylated proteins (Mancera-Arteu et al., 2020). Gao et al., 2019a reported a strategy to isolate serum exosome based on TiO_2_ microspheres. They compare serum exosome proteins from pancreatic cancer patients and healthy donors, identifying 59 significantly upregulated proteins (Gao et al., 2019a). Researchers also achieved the rapid enrichment of EVs from the serum using TiO_2_ magnetic beads to further recognize the EVs expressing PD-L1, supporting the TiO_2_ good ability for enriching EVs (Peng et al., 2020). Xiang et al., 2021 combined ultrafiltration and TiO_2_ to enrich EVs from many urine samples and conducted liquid chromatography–mass spectrometry (LC–MS) to identify proteins (Xiang et al., 2021). Dao et al. (2022) synthesized chimeric nanocomposites of lactoferrin conjugated 2,2-bis (methylol) propionic acid dendrimer-modified magnetic nanoparticles (LF-bis-MPA-MNPs) to isolate the EVs by electrostatic interaction, physical absorption, and biorecognition. The mechanism of EV isolation by LF-bis-MPA-MNPs is shown in Figure 6. The metallic oxide-based isolation method has many significant advantages for EV separation. First, the high affinity makes the incubation time concise. Second, the EVs can be separated by magnetic separation only after combining with magnetic beads. The EVs have a high recovery rate and easy operation and do not require many samples to meet downstream analysis requirements. It is expected to be used in POCT for EVs in the future by combining with other detection methods.

#### 4.1.9 Absorbent polymer-based method

**FIGURE 7:**
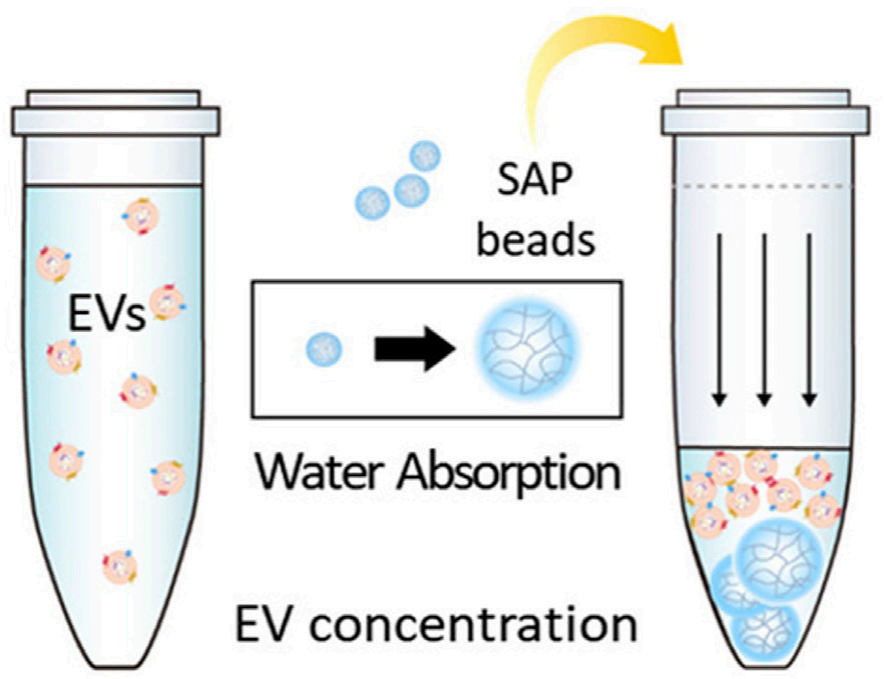
EVs enrichment *via* SAP bead. Small molecules are sucked into SAP with water, whereas EVs and other large size particles are excluded for concentration and purification. Reprinted with permission from Yang et al. (2021).

Super absorbent polymer (SAP) is a hydrogel with strong water absorption ability. There is a particular size space where relatively small molecules will be sucked into with water, while the EVs and other large size particles are excluded for concentration and purification. Some researchers developed a method to concentrate the EVs by SAP beads and successfully enriched the EVs from culture medium and urine (Yang et al., 2021). The principle is shown in Figure 7, where the method is simple and efficient without special equipment that can be used in the separation and purification of EVs in large volume samples, such as cell supernatant or urine. Compared with polymer co-precipitation, ultrafiltration and other methods, the method has advantages such as shorter concentration time and lower cost. However, the purity of EVs obtained is low and there is protein contamination with particle sizes similar to those of EVs. It is difficult to be directly applied to complex samples, such as blood. This method is simple to operate and highly scalable, which can be widely used in therapeutic or diagnostic applications that require EV enrichment.

There are two main aspects of application of EV-related miRNAs: one is the study on mechanism of action for target cells and the other is the detection of potential markers for disease. The impact of free protein and free protein-bound miRNA contamination on downstream experiments should be considered, as well as cost, flux, and automation. However, the tolerance of different experimental purposes and influence weight of related factors are different. For different applications, we can use the most suitable separation method, and the advantages and disadvantages of different isolation methods for application of EV-related miRNAs are as in Table 1.

**TABLE 1:** Summary of EV isolation methods.

Isolation method	Isolation mechanism	Advantages	Disadvantages	References
Ultracentrifugation	Density	Gold standard method, widely used	EVs yields are low	Thery et al. (2006), Lobb et al. (2015), Nigro et al. (2021)
			Long process	
Density gradient centrifugation	Density	High purity	Cumbersome operation; time-consuming	Ashley et al. (2018)
Size exclusion chromatography	Particle size	Used for large-scale samples	Each consumable can only handle samples from the same source, which is too costly if used for the separation of different clinical samples	(Boing et al. (2014), Guo et al. (2021)
Ultrafiltration	Particle size	The separation steps are simple and fast	Cannot remove similar-sized protein particles	(Haraszti et al. (2018), Li et al. (2019), Chen et al. (2021)
Co-precipitation	Surface charge	Simple and fast separation steps	Contamination of organelle-related proteins, not conducive to downstream detection	Rider et al. (2016), Ludwig et al. (2018)
Immunoaffinity enrichment	Antigen–antibody	Obtain EVs expressing specific proteins	Bind to free proteins and affects the capture efficiency of EVs; low recovery	Choi et al. (2021)
Field flow fractionation	Molecular weight	Wide range of separations	Special equipment; low-throughput	Zhang and Lyden, (2019)
Acoustic-based isolation method	Sound wave	The separation steps are simple and fast	Not suitable for complex samples	Lee et al. (2015b), Tayebi et al. (2021)
Metallic oxide-based isolation method	electrostatic interaction, physically absorption and biorecognition	Fast and simple; small sample volume; Low cost	Not conducive to downstream detection	Gao et al. (2019a), Dao et al. (2022)
Absorbent polymer-based method	Particle size	High-efficiency, No special equipment	Low purity; not suitable for complex samples	Yang et al. (2021)

### 4.2 Extraction and purification methods for microRNA

There are different isolation methods for EV, and there are also different separation and purification methods for miRNA (Chomczynski and Sacchi, 2006; Rio et al., 2010; Duy et al., 2015). The core of RNA separation is to improve the purity of extracted EV-derived miRNA and reduce the losses (Lin et al., 2022). TRIzol method can extract miRNA from the EVs. TRIzol is a reagent whose main component is phenol that can maintain RNA integrity during sample lysis or homogenization. After adding chloroform, the solution is divided into aqueous and organic phases. RNA can then be precipitated in the aqueous phase with isopropyl alcohol (Burgos et al., 2013). But the main issue with precipitation-based isolation is the incomplete removal of phenols and salts. Some miRNAs may be lost during the extraction (Kim et al., 2012), but the reagent chloroform is dangerous. The TRIzol method accompanied by column purification (Brown et al., 2018) has high efficiency. Using the solid phase to isolate the EV miRNA, such as magnetic beads (Xu et al., 2018; Mahmudunnabi et al., 2022), can make the process simple. The high salt concentration is also necessary to solve, which can influence the downstream analysis. McAlexander and fellow researchers (McAlexander et al., 2013) compared different RNA extraction methods and drew the conclusion that not all extraction methods are good. RNA extraction from different sources refers to different extraction methods. Therefore, a fast and simple EV-derived miRNA extraction method is needed.

## 5 Advances in detection of extracellular vesicles–derived microRNA

Current techniques for qualitative and quantitative analysis of miRNAs in samples have developed. Quantitative reverse transcription polymerase chain reaction (qRT-PCR) based on amplification is the typical method for miRNA detection in EV. Furthermore, many other strategies have been established, including molecular beacon *in situ* (MB *in situ*), surface-enhanced Raman scatting (SERS), microarray, next-generation sequencing (NGS), and isothermal amplification. Except NGS, the above methods can detect specific miRNAs by probe or primer that is specifically complementary to the target miRNA.

### 5.1 Quantitative reverse transcription polymerase chain reaction

Currently, the most widespread application for detection of extracellular vesicle miRNA is qRT-PCR (Drula et al., 2020), which is regarded as a gold standard for the detection of gene expression. Quantification of RNA molecules by qRT-PCR unusually consists of two steps: first, the complementary DNA with RNA targets is synthesized by reverse transcriptase (RT), and then PCR amplification is performed using cDNA as a template. The amplification process is monitored in real time by detecting the fluorescence of specific dye of double-stranded DNA or a specific fluorescent probe. Currently, there is no accepted internal reference for EVs that is commonly used in cellular miRNA, such as gene U6 is not stably expressed in the EVs; therefore, it is crucial to find the stably expressed internal reference genes, which is suitable for samples before analyzing the miRNA. Fang et al. (2021) designed a portable nucleic acid detection (PNAD) system and realized the sample processing and PCR in a single device. Compared to traditional PCR techniques, droplet digital PCR (ddPCR) makes a technical improvement, solving the problem of sensitivity in detecting low copy number of transcripts (Hindson et al., 2013). The reaction system was divided into thousands of oil droplets, which encapsulated all reagents, but only one copy of the template was amplified. The fluorescence signal from each droplet was measured by the ddPCR instrument. ddPCR has higher accuracy and reproducibility for serum miRNA (Hindson et al., 2013). Moreover, researchers have recently shown that the ddPCR has higher sensitivity, repeatability, and accuracy in detecting miRNA in urine exosomes, compared with qPCR (Wang et al., 2019). The ddPCR is sensitive to detecting nucleic acids with low copy numbers, but it requires special instruments and costs highly. Moreover, Zhou et al. (2021) detected the EV-derived miRNA in the plasma of patients with endometrial cancer (EC) by PCR and found that mir-15a-5p, mir-106b-5p, and mir107 were significantly upregulated in the exosomes isolated from plasma samples of EC patients, compared with healthy controls.

### 5.2 Molecular beacon *in situ*

A series of situ detection methods for miRNAs in exosomes were developed by molecular beacon (MB) (Zhai et al., 2018; Oliveira et al., 2019; Mao et al., 2022). The MB is able to bind to target RNA specifically, resulting in failure of quenching group and generation of fluorescence. The fluorescence intensity has a direct correlation with concentration of hybridizing molecular beacon with miRNA (Lee et al., 2018). These methods neither need RNA extraction nor amplification process, showing characteristics of simple operation. They only need the incubation of the sample with the beacon without removing the unbound beacon, but there is a false negative problem caused by low concentration (Lee et al., 2015a; He et al., 2019). Gao et al. (2019b) have reported a virus-mimicking fusogenic vesicle packaging MB by membrane disposal MB hybridized with target miRNAs in the exosomes for *in situ* detection.

### 5.3 Surface-enhanced Raman scatting

**FIGURE 8:**
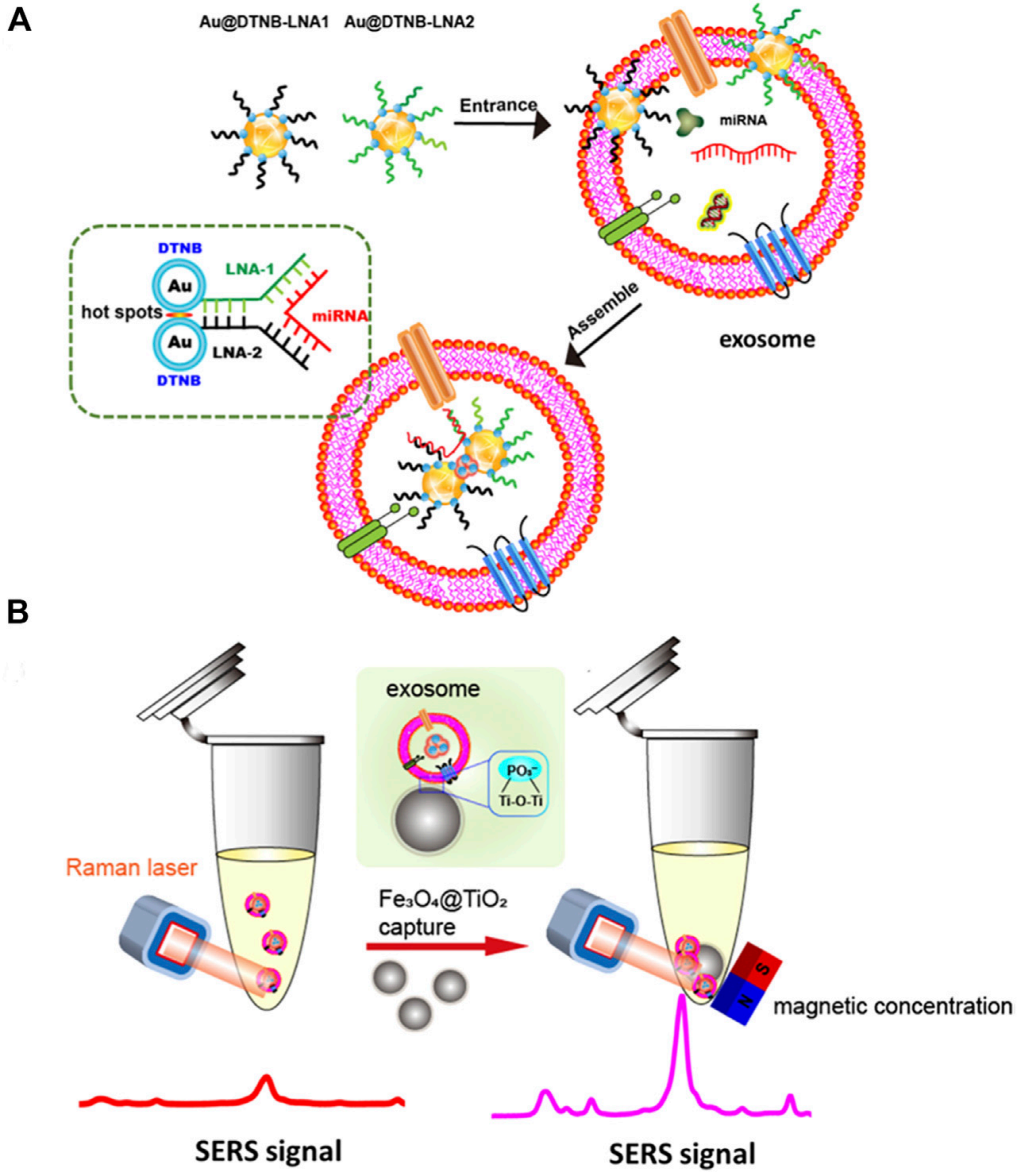
Exosomal miRNA *in situ* detection using SERS biosensor combined with Fe_3_O_4_@TiO_2_-based concentration. SERS tags are adsorbed to exosomes and bind to the target exosomal miRNA **(A)**. The hot spots and SERS intensity increase, and SERS intensity increases as Fe_3_O_4_@TiO_2_ captures exosome for *in situ* detection of exosomal miRNAs **(B)**. Reprinted with permission from Jiang et al. (2021a).

Surface-enhanced Raman scatting (SERS) can stimulate signals from small molecules on the metal surface (Kneipp et al., 1996). It can also be used for miRNA detection and classification and have several advantages, such as high sensitivity for detecting very low-level analytes with specific molecular fingerprints (Driskell et al., 2008). More and more research focus on detection of EV-derived miRNA using SERS. A recent study used a modified dual SERS biosensor to detect miRNAs isolated from exosomes. Biosensor uses the SERS label of Fe_3_O_4_@Ag-DNA-Au@Ag@DTNB that possesses DNA oligonucleotides complemented with target miRNA. Target miRNA hybridizes with DNA oligonucleotides on nanoparticles labeled SERS, and then DNA from the miRNA/DNA duplexes is selectively cleaved by duplex-specific nuclease. Cutting causes the separation of SERS label from the substrate, and the quenching reaction can be detected and low detection limit of 1 a.m. copy number can be achieved (Pang et al., 2019). Researchers reported an *in situ* platform for directly detecting exosomal miRNAs from serum samples (Jiang et al., 2021a). They used Au@DTNB-modified [DTNB is the Raman reporter molecule 5,5′-dithiobis-(2-nitrobenzoic acid)] locked nucleic acids (LNAs) to introduce into exosomes and bind to targeted miRNAs to generate SERS signals. The SERS signal is enhanced by Fe_3_O_4_@TiO_2_ enrichment of exosomes. As shown in Figure 8, target miRNAs can be directly qualified *in situ* with a detection limit of 0.21 fM based on the platform, which is better or comparable to qRT-PCR. The SERS detection has the advantages of being fast, simple, and inexpensive. More portable SERS detection equipment will appear in the future.

### 5.4 Microarray

Microarray is based on a predesigned label probe that hybridizes with the target cDNA sequence (Sevenler et al., 2018). Total RNA was herein extracted from the EVs isolated from the sample, and then a cDNA library that complements with RNA was constructed. To realize the detection of miRNA, cDNA is mixed up with the label probe fixed on the chip, and complementary sequence is hybridized with the labeled probe in the microarray, while the relative gene expression level is quantified according to the hybridization signal intensity (Li et al., 2020). Microarray has been widely used in the analysis of miRNA in EVs. Nakamaru et al. (2020) analyzed the expression of EV-derived miRNA in type 1 autoimmune pancreatitis (AIP) samples, chronic pancreatitis (CP), and healthy adult (HC) samples by microarray technology and found that mir-21-5p was highly expressed in AIP compared with control group. Roman-Canal et al., 2019 used TaqMan open array technology to analyze the expression of miRNAs related EVs and found that 210 miRNAs were differentially expressed in the EVs isolated from peritoneal lavage fluid of patients with colorectal cancer (CRC) (Roman-Canal et al., 2019). Although the miRNA chip can analyze a large number of sequences simultaneously; however, it has low sensitivity, narrow dynamic range, high cost, and extended operation.

### 5.5 Next-generation sequencing

NGS is a high-throughput sequencing method that is the leading technology in transcriptome analysis (Alexandra et al., 2022; Griffin et al., 2022). It can sequence the base pair of DNA or RNA. First, RNA in the sample needs to be extracted and purified. Universal adaptors are often connected to the 5′ and 3′ ends of each RNA strand, and then reverse transcription and PCR amplification, and finally, the products are sequenced. NGS technology overcomes some limitations of microarray to study miRNA in EVs, such as requiring many samples being unable to detect unknown miRNA, and has higher sensitivity and accuracy than microarray. NGS is usually used to screen the disease-related specific miRNA. In another study, researchers analyzed the noncoding RNA expression profile of prostate cancer metastatic cells (PC3) and EVs by NGS technology and found that miR-10a-5p and miR-29b-3p from the EVs in the plasma sample from patients with prostate cancer were significantly overexpressed (Worst et al., 2020). Sequencing technology can find the new gene sequences, but it is not suitable as the standard detection technology because of its long cycle, high cost, and complex data analysis.

### 5.6 Isothermal amplification technique

**FIGURE 9:**
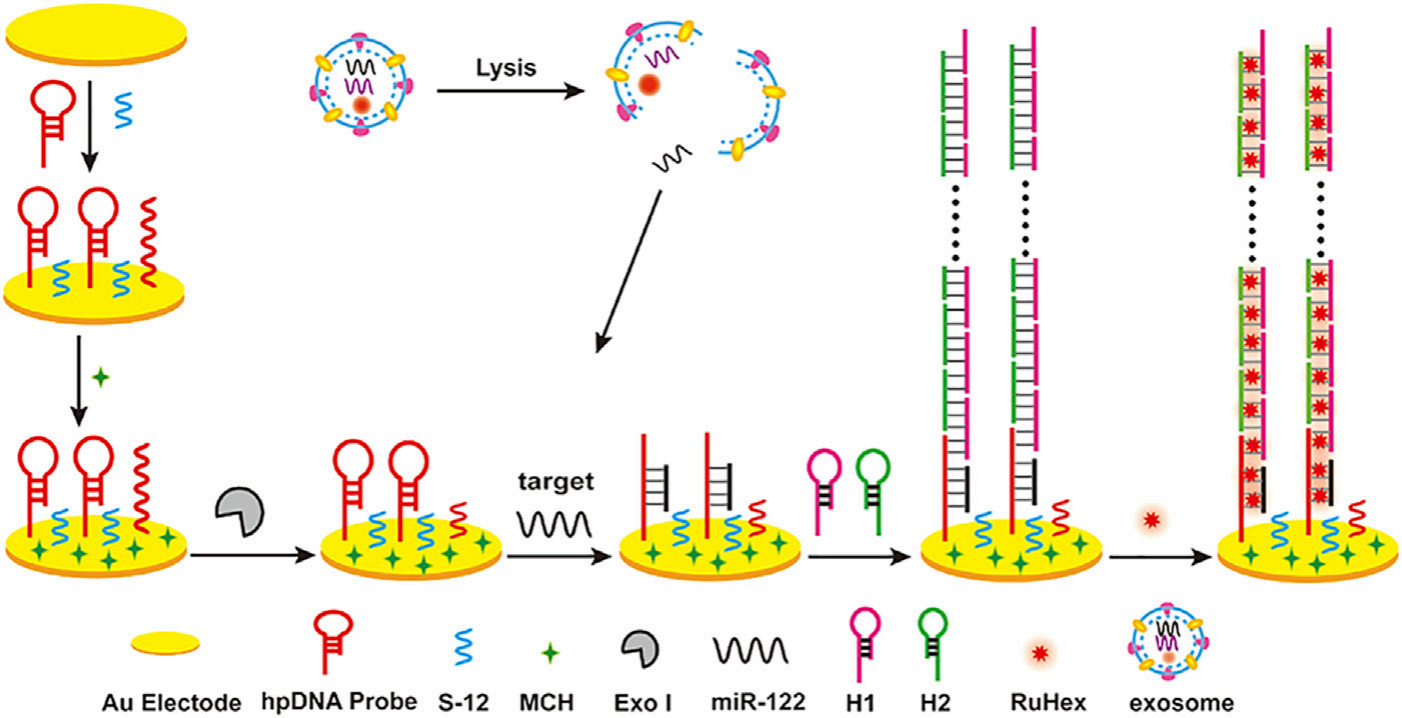
Exosomal miRNA detection by an electrochemical sensing platform harnessing HCR as a signal amplification strategy. Reprinted with permission from Guo et al. (2020).

In recent years, isothermal amplification has become a popular method for detecting miRNA (Zhao et al., 2015; Reid et al., 2018). It is easy for isothermal amplification methods to have allowed nucleic acid amplification to be carried out, and isothermal amplification has had a profound impact on the way molecular diagnostics operate (Glokler et al., 2021). Because it does not need a thermal cycler, isothermal amplification is more suitable for short-sequence RNA. MiRNA is used as the primer extended on the template, triggering sequence amplification or template for primer design (Deng et al., 2017). As described in the overviews (Gines et al., 2020), isothermal amplification is usually divided into enzyme and enzyme-free methods. Enzymatic isothermal amplification includes loop-mediated isothermal amplification (LAMP), nuclear acid sequence-based amplification (NASBA), rolling circle amplification (RCA), exponential amplification reaction (EXPAR), and duplex-specific nuclease amplification reaction (DSNAR). Recent studies show a sensitive detection method for miRNA in various exosomes using RCA technology to detect miRNA-21, miRNA-122, and miRNA-155 (Wang et al., 2021) in exosomes at the same time. Compared with conventional methods, it has the advantage of multiple detection and simplicity of RCA. Enzyme-free isothermal amplification methods for miRNA detection include catalytic hairpin assembly (CHA) and hybrid chain reaction (HCR) (Gines et al., 2020). Guo et al. (2020) developed a label-free sensitive electrochemical detection method based on HCR for signal amplification to detect miRNA-122 in exosomes (Figure 9), and the detection limit was as low as 53 a.m.

Table 2 shows the advantages and disadvantages of current detection methods for miRNA. With fast development of quantitative detection technology, the emergence of EV miRNA has become a particular and sensitive diagnostic and prognostic biomarker that is expected to be used for noninvasive detection of diseases.

**TABLE 2:** Summary of EV-derived miRNA detection methods.

Method	Time	Advantages	Disadvantages	References
RT-qPCR	>2 h	Gold standard method, high sensitivity	Time-consuming for heating, many other influencing factor	Jiang et al. (2021b)
ddPCR	>2 h	High sensitivity	Special equipment, unable to popularize	Wang et al. (2019), Zhou et al. (2021)
MB *in situ*	<1 h	Simple operation, no amplification, *in situ*	Low sensitivity	Lee et al. (2018)
SERS	∼1 h	Low cost and simple operation	High requirements for chip manufacturing, Low repeatability, special equipment, unable to popularize	Pang et al. (2019)
Microarray	∼2 days	Detect many sequences at once	High cost and time-consuming for a small number of samples, unable to popularize	Nakamaru et al. (2020), Tan et al. (2021)
NGS	3–7 days	Find unknown miRNA	Time-consuming and high cost	Selmaj et al. (2017)
Enzymatic isothermal amplification	>1 h	High amplification efficiency without heating	Complex primer design, not easy to realize multiple detection	Wang et al. (2021)
Enzyme free isothermal amplification	>2 h	No enzyme, low cost	Complex primer design	Guo et al. (2020)

## 6 Summary and future prospect

In this review, we have discussed the mechanisms for miRNA sorting in EVs and diagnostic value of EV-derived miRNA in diseases. We then summarized the isolation methods for EVs and EVs’ miRNA-related detection technologies. Moreover, we also illuminated the advantages and disadvantages of each method, that can be valuable for deciding the most suitable isolation and detection methods in different situations.

Ample progress has been made in studies on EV-derived miRNAs in a plethora of diseases, but there are still many challenges with unclear mechanisms. However, the current EV-derived miRNA extraction technology still has many challenges to solve. First, the inefficient EV separation method may lead to the loss of many EVs, affecting the downstream analysis of EVs contents. Second, the EV-derived miRNA extraction refers to extraction method for miRNA from cells and body fluids. For example, the TRIzol method is widely used to extract nucleic acids, but it has limitations such as complicated operation and high miRNA loss, which hinder the application of EV-derived miRNA for clinical diagnosis.

Many disease-related EV miRNAs have been discovered, and detection of EV miRNA has made significant progress and is now close to commercial application. It has significant social value and economic benefits to develop a new method for automatic, rapid isolation of EVs and extraction of miRNA in large samples. In the aspect of EV isolation, the method can be improved to get EVs with high purity by employing various methods, such as using magnetic beads or microfluidics technology. For the miRNA detection, the application of multiple detections of different EVs’ miRNA and detection of EV miRNA in combination with EV-related membrane proteins, DNA, or glycoproteins can improve the sensitivity and specificity of the detection methods. Detection of miRNA from a single EV ensures the integrity of the EV structure and information without extraction of RNA, which is the direction for future development. For some EV-derived miRNAs associated with acute diseases, the development of simple, rapid, and sensitive miRNA extraction and detection POCT technology is also one of the future research directions.
